# Informed Consent for Ambient Documentation Using Generative AI in Ambulatory Care

**DOI:** 10.1001/jamanetworkopen.2025.22400

**Published:** 2025-07-22

**Authors:** Katharine Lawrence, Vasudev S. Kuram, Defne L. Levine, Sarah Sharif, Conner Polet, Kiran Malhotra, Kellie Owens

**Affiliations:** 1 Department of Population Health, New York University Grossman School of Medicine, New York; 2 Department of Health Informatics, Medical Center Information Technology, NYU Langone, New York, New York

## Abstract

**Importance:**

Artificial intelligence (AI)–assisted ambient documentation technologies that use audio from clinic visits to generate documentation are being deployed across hospital systems to optimize clinical note writing and reduce administrative burden. However, little is known about optimal approaches to engage patients in informed consent for these tools.

**Objectives:**

To explore clinician and patient experiences with consent processes and examine the association between the use of ambient documentation tools and the patient-clinician relationship.

**Design, Setting, and Participants:**

This study was conducted from March 1 to December 31, 2024, in ambulatory practices across specialties in a large urban academic health center as part of an ongoing operational quality improvement initiative. Participants included clinicians and patients participating in an operational proof-of-concept exploration of ambient documentation technology.

**Main Outcomes and Measures:**

A pragmatic, sequential, inductive-deductive qualitative evaluation was conducted of informed consent contexts, processes, and challenges among ambulatory clinicians and patients exposed to ambient documentation technology. Evaluation included site visits, clinical observations, clinician interviews, and patient surveys to explore perceptions and challenges related to consent.

**Results:**

A total of 121 ambient documentation pilot users included 18 clinicians (mean [SD] years of practice, 18.6 [100]; 10 men [55.6%]) and 103 patients (mean [SD] age, 37 [12.5] years; 65 women [63.1%]). The most common consent approach was a verbal patient-clinician conversation prior to an individual encounter. Patients and clinicians had a spectrum of comfort with ambient technology; 77 patients (74.8%) reported being comfortable or very comfortable with their physician using ambient documentation. Patient trust, detail in the consent discussion, and intended tool use were associated with patient comfort and intent to consent. Technical understanding was associated with comfort with consent conversations: when provided basic information about the technology, 84 patients (81.6%) consented; this decreased to 57 patients (55.3%) when details about AI features, data storage, and corporate involvement were disclosed. Perceived benefits included reduced documentation burden, improved decision-making, and enhanced communication. Concerns included data security, legal liability, cognitive impacts, and equity. When asked about responsibility for medical errors linked to ambient documentation, 66 patients (64.1%) held physicians accountable; for data security breaches, 79 patients (76.7%) believed vendors should be responsible. Participants suggested a flexible consent model with digital touchpoints, education, nonclinical staff support, and opt-out options.

**Conclusions and Relevance:**

In this quality improvement study of 121 pilot users of AI-assisted ambient documentation technology, informed consent relied primarily on verbal conversations that varied based on time, knowledge, and the patient-clinician relationship. A flexible, multimodal approach—including education, time to discuss risks and benefits, digital resources, nonclinical staff involvement, and clear opt-out options—may improve consent processes and support broader acceptance of ambient documentation tools.

## Key Points

**Question:**

How do patients and clinicians experience and navigate informed consent processes for ambient documentation using generative artificial intelligence in outpatient care?

**Findings:**

In this quality improvement study involving 121 users (18 clinicians and 103 patients) at a large urban academic health center, patient comfort varied based on trust in their clinician, understanding of the tool, and perceived benefits or risks. Clinicians prioritized workflow efficiency, risk mitigation, and communication clarity during consent conversations.

**Meaning:**

This study suggests that the informed consent conversation is a critical but nuanced touchpoint that shapes the adoption and acceptance of technology by both clinicians and patients; flexible, multimodal approaches that include education, digital tools, and opt-out options may enhance engagement with ambient documentation.

## Teaser

This quality improvement study explores clinician and patient experiences with informed consent processes for ambient clinical documentation using generative artificial intelligence (AI).

## Introduction

Since the release of OpenAI’s ChatGPT in November 2022, health care systems have been working to integrate large language models and other generative artificial intelligence (AI) tools into clinical workflows to improve efficiency and patient outcomes. One such application is AI-supported ambient clinical documentation, which uses audio from clinic visits to generate electronic health record documentation. Such products aim to reduce administrative burdens, giving clinicians more time to engage with patients.^1,2^

Ambient documentation has the potential to enhance patient encounters by allowing clinicians to focus more on direct, empathetic interactions, strengthening trust and rapport. However, its introduction raises ethical, privacy, and equity concerns^3,4^ that could affect patient-clinician relationships.^5,6^ Risks include confidentiality issues that may lead to censored conversations and barriers to equitable use,^3^ such as challenges in non-English encounters and multiparty visits, as well as for those with limited digital health access.^5,7,8,9,10^

The rapid deployment of AI-assisted ambient documentation highlights critical ethical questions about its responsible use in patient care. A key concern is informed consent, a legal and ethical requirement based on patient autonomy.^11^ Core elements of consent include disclosure, comprehension, voluntary choice, and authorization, and obtaining informed consent is a legal, ethical, and regulatory requirement for most health care delivery.^11^ However, informed consent practices vary in practice, and the process may be overlooked when integrating new technologies into routine care.

Although previous studies have explored patients’ perspectives, values, and concerns associated with the use of AI technologies in clinical care, there is limited research exploring the unique contexts of AI tools that passively record information for clinical documentation purposes (ambient documentation) and how patients and clinicians navigate conversations and decision-making about the use of these tools. This study explores clinician and patient experiences with the consent process for AI-assisted ambient documentation, its limitations, and the contextual and informational needs necessary for informed discussions. We also examine how decisions to use or decline the use of these tools are associated with the patient-clinician relationship.

## Methods

### Study Approach and Analysis

To explore the ethical considerations surrounding informed consent for the use of ambient documentation in patient visits, we conducted a qualitative evaluation of 121 ambient documentation pilot users (18 ambulatory clinicians and 103 patients) centering on informed consent contexts, processes, and challenges at a large urban academic health center in New York City. This was an observational study of a quality improvement initiative focused on the implementation and evaluation of ambient AI documentation tools. At the outset, we anticipated that participating clinicians would be involved in testing and providing feedback on the technology to inform the ongoing optimization and implementation strategy. Based on this intent, the work met the definition of quality improvement as defined by the NYU Grossman School of Medicine institutional review board and was determined not to constitute human subjects research; therefore, institutional review board approval was waived. An institutional review board–approved quality improvement self-attestation was completed by the study team. Individual verbal consent to use the technology was obtained at the patient level by clinicians at the start of each visit, consistent with NYU Langone Health patient care policies.

We used data triangulation approaches to collect information from multiple sources, including online clinician surveys, in-person interviews, clinical observations, email exchanges with clinicians and research staff, and project team notes and meeting minutes. A convenience sample of 34 clinicians who had participated in an early operational pilot of ambient AI documentation were contacted via email between March 1 and December 31, 2024, to request site visits and shadowing opportunities; 18 clinicians agreed to participate. Participating clinicians were observed in ambulatory clinic settings while using the ambient documentation tools. Observations included instances in which clinicians introduced the tool to patients and obtained verbal consent to use ambient AI technology during the visit, as per institutional practice. Observers documented real-time workflows and environmental factors that were associated with consent conversations. Clinicians were also interviewed in person immediately after observation sessions to gather reflections on their own experiences with consent processes, their understanding of institutional policies, and their perceptions of patient comfort (eAppendix 1 in Supplement 1). To incorporate patient perspectives but preserve patient privacy and security, we partnered with the commercial user research platform Dscout,^12^ a qualitative research tool that enables researchers to recruit and survey participants through an app-based interface; for this study, we screened for NYU Langone Health patients who reported prior experience with ambient documentation tools during their outpatient visits and expressed a willingness to provide anonymous feedback. The Dscout platform obtained participant consent via its standard opt-in enrollment process, which includes agreement to participate in research activities. A total of 103 patients completed a 60-minute survey that included both closed- and open-ended questions about their actual experience with ambient documentation, as well as their attitudes and concerns about the broader implications of such technologies (eAppendix 2 in Supplement 1). Patient feedback included both descriptive accounts of ambient documentation experiences and responses to hypothetical scenarios related to transparency, privacy, data use, and consent processes. Participants received a $30 honorarium for their time.

### Statistical Analysis

We used a sequential inductive-deductive approach to collect and analyze data—inductive methods enabled discovery and exploration, while deductive methods provided structure and validation. The inductive phase included site visits, observations, and informal clinician and patient feedback on an ambient documentation product, as part of a larger initiative evaluating its effect in ambulatory practice. This was supplemented by a literature review of academic and trade sources, vendor materials, conference proceedings, and internet forums (eg, Epic UserWeb). Investigators (K.L. and V.S.K.) conducted open qualitative coding to identify themes. Based on these findings, we conducted clinician interviews (virtual and in person) and patient surveys (via DScout User Research) to examine perceptions of informed consent. Focused coding explored gaps in consent processes, responsibility for discussions, and clinician and patient knowledge needs.

## Results

The demographic characteristics of the 121 participants are detailed in Table 1 (patients) and Table 2 (clinicians). Of 103 total patients (mean [SD] age, 37 [12.5] years), 65 (63.1%) self-identified via survey as women, 36 (34.9%) self-identified as men, and 2 (1.9%) identified as nonbinary or other gender (Table 1). Most patients were college graduates (60 [58.3%]) or had postgraduate education (24 [23.3%]), had employer-based health insurance (72 [69.9%]), and were English speaking (95 [92.2%]). A total of 16 patients (15.5%) identified as Asian, 16 (15.5%) as Black or African American, 13 (12.6%) as Latine, 46 (44.7%) as White, and 12 (11.7%) as other race or more than 1 race.

**Table 1.** Self-Reported Patient Demographic Characteristics

Demographic characteristic	Patients, No. (%) (N = 103)
Gender identity	
Woman	65 (63.1)
Man	36 (34.9)
Nonbinary or other	2 (1.9)
Race and ethnicity	
Asian	16 (15.5)
Black or African American	16 (15.5)
Latine	13 (12.6)
White	46 (44.7)
Other or >1 race and ethnicity^a^	12 (11.7)
Age, y	
18-39	35 (34.0)
40-59	30 (29.1)
60-69	3 (2.9)
Prefer not to say	35 (34.0)
Highest educational level	
Some high school	2 (1.9)
High school graduate	5 (4.9)
Some college	12 (11.7)
College graduate	60 (58.3)
Postgraduate coursework	24 (23.3)
Household income, $	
<25 000	6 (5.8)
25 000-49 999	11 (10.7)
50 000-74 999	12 (11.7)
75 000-99 999	18 (17.5)
≥100 000	49 (47.6)
Prefer not to say	7 (6.8)
Type of health insurance	
Employer	72 (69.9)
Private	9 (8.7)
Public health insurance (eg, Medicare)	22 (21.4)
English as first language	
Yes	95 (92.2)
No	8 (7.8)

^a^ Patients who selected “Other (please specify)” included the following: American Indian or Alaska Native, Guyanese, Jewish, Middle Eastern or North African, multiple races, Native Hawaiian or other Pacific Islander, Polish, prefer not to say, Romany, and Russian.

**Table 2.** Clinician Demographic Characteristics Plus Practice Information, Based on Publicly Available Practice Information

Demographic characteristic	Clinicians, No. (%) (N = 18)
Gender	
Woman	8 (44.4)
Man	10 (55.6)
Nonbinary or other	0
Specialty	
Internal medicine or primary care	13 (72.2)
Medicine subspecialty	3 (16.7)
Surgical subspecialty	2 (11.1)
Years in practice	
<5	2 (11.1)
5-10	3 (16.7)
>10	13 (72.2)
Practice location	
Manhattan	8 (44.4)
Brooklyn	4 (22.2)
Queens	1 (5.6)
Other	5 (27.8)
Language other than English spoken	4 (22.2)

Of 18 clinicians (mean [SD] years of practice, 18.6 [10] years), 10 (55.6%) identified as men and 8 (44.4%) identified as women via self-report or public review of demographic information (Table 2). Thirteen clinicians (72.2%) specialized in internal medicine or primary care, while 5 clinicians (17.2%) specialized in another medicine or surgical subspecialty. Most clinicians (13 [72.2%]) had been practicing for more than 10 years.

We identified the following factors associated with experiences with and preferences for consenting to AI-assisted ambient documentation: patient and clinician comfort with ambient documentation technology, assessment of benefits and risks of ambient tools, and perceptions of responsibility for ambient tools. We also identified challenges, preferences, and opportunities for optimizing the consent process for ambient documentation.

### Patient and Clinician Comfort With Ambient Documentation Technology

Patients expressed varying degrees of comfort with ambient documentation technology based on their views on technology, data policies, and medical contexts. Overall, 77 patients (74.8%) reported being comfortable or very comfortable with their physician using ambient documentation. Despite this, most voiced concerns about data confidentiality, storage, and trust in the handling of sensitive information (Box 1). A total of 61 patients (59.2%) did not want their data shared with the vendor of the ambient documentation technology.

Box 1. **Representative Quotes for the Theme of Patient and Clinician Comfort With Ambient Documentation Technology**

**Varying general comfort**

I’m okay with my doctor trying out new technology as long as it doesn’t involve selling personal data to third parties. [Patient]
I’m pretty uncomfortable with a for-profit AI company having access to my audio visits. [Patient]
Using AI in this way with a for-profit company is completely unacceptable. I would never see a doctor who uses this. [Patient]
It’s a tool, I don’t think of it any other than a tool, just used for documentation. [Physician, internal medicine]
I feel not that different [about AI] than any other technology. [Physician, internal medicine]
Unclear on what it means for everything that happens in the room to be captured, it’s a bit creepy. [Physician, internal medicine]
When you are being recorded, you’re going to be more careful about what you say—that’s the Hawthorne effect. [Physician, medicine subspecialty]

**Clinical scenario and uses**

If I tell my doctor my private problems and he records it, I would feel violated unless… the conversations on that device can be filtered/deleted. [Patient]
I would also probably self-censor. [Patient]
In this political climate it’s even more important to protect health information for women. I would be terrified that this information could be gathered and used against me. [Patient]
I would prefer this tool to create a transcript of the conversation for records. I don’t love the idea of the tool diagnosing me, but I do think it’s useful to capture details of what was said. [Patient]
I think the ultimate judgement and diagnosis should still have its final decision made by a doctor and it should merely be a tool that complements the existing visit. [Patient]

**Data policies and practices**

I would ask where this is archived, his office only or some outside source, and how comfortable doc feels about who sees the information. I won’t even use “health apps” because you don’t know what the company does with that information. [Patient]
It’s not that I think the recorded information would get leaked, more that the information will permanently be on record somewhere. [Patient]
I would need written agreement or contract that my information would not be leaked or shared to others. [Patient]
I wondered where the data was being stored. When the [product team] told us it would be stored up to a year at some place and they would study the transcript to make the [technology] better, that didn’t make me comfortable. [Physician, internal medicine]

**Trust, patient-clinician relationship**

I trust my doctor, and I am sure he’d be using the technology to help better serve me during my visit. [Patient]
I trust my doctor. I also know what a big role technology plays in all of our lives these days… It seems reasonable to me for my doctor to use the latest technology to not only make her job easier, but also to be able to provide me, the patient, with even more of her attention and focus. [Patient]
I trust my doctor and the hospital network that she is affiliated with is one of the best in the country so I would feel that if they are using the technology that it has been tested rigorously and approved by the board, lawyers, compliance specialists, HIPAA specialists, etc. [Patient]
I don’t know if it changes all that much, a visit is a visit… I have strong relationships with my patients, I’m there twice a week… Everyone there is people I’ve seen for 20 years. [Physician, internal medicine]
It will only increase trust, because they would appreciate the human piece even more. Trust building is very important. [Physician, medicine subspecialty]
Patients say: anything they can do to make it easier for you (doctor), they are ok with it. [Physician, internal medicine]
I have good rapport with my patients, they say “Anything to help you, Doc.” [Physician, internal medicine]

Abbreviations: AI, artificial intelligence; HIPAA, Health Insurance Portability and Accountability Act.

Patient comfort with ambient documentation varied by clinical scenario, affecting intent to consent. Patients were most at ease with ambient documentation for routine physical examinations, with 65 patients (63.1%) reporting no change in behavior if the technology was used in this context. However, patients were more likely to censor what they said when discussing mental health (36 [35.0%]), sexual health (42 [40.8%]), or illicit activity (53 [51.5%]). Patients also preferred ambient documentation for routine note generation (65 patients [63.1%] comfortable with this use) compared with AI-supported clinical reasoning in treatment plans (44 patients [42.7%] comfortable with this use) or diagnosis (31 patients [30.1%] comfortable with this use) (Box 1). In addition, patients discussed their discomfort sharing personal information relevant to larger sociopolitical events associated with potential risks of the sensitive data being used against them (Box 1); 80 patients (77.7%) wanted to know if discussing illegal activity in a recorded encounter carried legal risks.

Clinicians’ framing of consent conversations was associated with patient comfort. When provided basic information about the technology (Figure, A), 84 patients (81.6%) consented; this decreased to 57 patients (55.3%) when details about AI features, data storage, and corporate involvement were disclosed (Figure, B).

**Figure.**
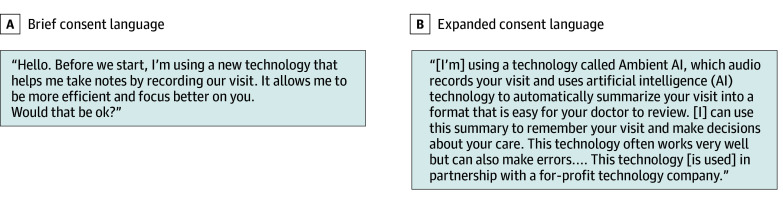
Sample Consent Language: Brief and Expanded

Compared with patients, clinicians expressed similar concerns regarding the use of ambient documentation data. Many were unsure about how data were stored or secured and wanted more information about data practices prior to using the technology with patients (Box 1). They also worried about medico-legal risks, including liability and discoverability. Some were concerned that AI might capture unaddressed complaints, leading to missed diagnoses or legal exposure, while others reported heightened accountability knowing that every interaction was recorded.

Among patients and clinicians, trust was identified as a central factor in patients’ willingness to consent to the use of ambient documentation. Patients cited trust in their clinician, health care system, and industry as influencing their decision to agree to ambient technology use (Box 1). Clinicians acknowledged the tool’s potential effect on relationships with patients—most felt that the rapport remained unchanged, but some noted that patients might withhold sensitive information when the tool was present. Some clinicians reported selective use of the ambient documentation tool based on a variety of factors, including preexisting relationship dynamics and knowledge of the patient. In these settings, clinicians reported using a “gut feeling” to identify “the patients who won’t like it.”

### Assessing Benefits and Risks of Ambient Documentation Tools

Patients and clinicians shared perspectives on the benefits and risks of ambient documentation in clinical encounters. Perceived benefits included reduced documentation burden, improved recall, and enhanced communication and rapport. Risks involved data security, legal liability, cognitive impacts, and equity concerns.

Patients generally valued the potential of ambient documentation to ease clinicians’ notetaking burden and improve care delivery (Box 2). Some noted its ability to enhance memory and recall, supporting medical decision-making. Others hoped it would improve clinician-patient relationships and overall care experiences (Box 2).

Box 2. **Representative Quotes for the Theme of Assessing Benefits and Risks of Ambient Tools**

**Reducing documentation burden and improving clinician work (benefit)**

If it helps my doctor actually write notes better as opposed to the horrific handwriting he currently has, then maybe he finds or remembers something that might help. [Patient]
It is not necessarily a bad thing. It can aid, and if it makes their job easier, why not? [Patient]
I don’t have to sit there and try to jot down notes, I can spend time reviewing X with patients and giving time with patients. [Physician, internal medicine]
I need it. I welcome it… I am not a good typist and it is terrible that I am typing out the note because it is not easy. I would welcome this function. [Physician, internal medicine]
I think it’s certainly helping us become more efficient clinicians and will be refined and improved over time. [Physician, internal medicine]
In medicine, we absolutely need AI. There is so much information now available to me that I am responsible for. In the EHR and all the clinical knowledge I am supposed to know. [Physician, internal medicine]

**Communication, patient-clinician relationship, patient experience (benefit)**

I like the idea of AI summarizing and notating audio in order to pay more attention to me. That would help both me and my doctor immensely. [Patient]
Medicine is all about embracing new practices and technology. There is transparency in addressing it upfront and it would only help to create a better patient experience. [Patient]
If I’m looking at the monitor, the patient is off to my left and may be sitting at the edge of the table where my body is even with them but I’m looking a completely different direction. Now I’m taking a step back and looking at them directly. That’s an improvement. [Physician, internal medicine]
I feel forced to speak out loud a lot. It’s probably for the better. When you do a physical exam, it is nice to voice things because otherwise you may just move on and patients have no idea if everything is ok. [Physician, internal medicine]
I said I would show them the note it produced, and I think people liked that. [Physician, internal medicine]

**Data policies and practices (risk)**

Where is the conversation going, will anyone else hear it, or how are you going to use this to help you? [Patient]
Listen, personal information is A BIG deal for me and others, if I tell my doctor my private problems and he records it, I would feel violated UNLESS like ChatGPT the conversations on that device can be filtered/deleted. [Patient]
I would need written agreement or contract that my information would not be leaked or shared to others. [Patient]
Doctor-patient relationship is very private so where do we go when someone says something private—where does it go with the use of AI. [Physician, internal medicine]
Just the idea that the audio is being recorded to my phone can be scary for [my patients]. [Physician, internal medicine]

**Medical decision-making and medico-legal issues (risk)**

A huge part of our learning is active learning, the synthesis part. I am assembling symptoms and as I am writing them down, part of my brain is learning and synthesizing it into an illness script. [Physician, internal medicine]
It’s functioning as a dictation service but also interpreting what is spoken about but it has given a patient a diagnosis that doctor doesn’t think he has. [Physician, internal medicine]
Negative impact could be that people review notes less carefully so the notes could become more garbage-y. [Physician, internal medicine]
You are putting a human being in a situation where they are expected to be perfect… like a computer. Where I remember every little thing. That sounds horrible. [Physician, internal medicine]
If the AI says every single complaint and you didn’t address one, then you may be held liable… [Physician, internal medicine]

**Equity (risk)**

[I had] one patient with a heavy [accent], and he asked “Will this understand me?” And I said, “You know, I don’t know.” So, we didn’t use it. I didn’t want to put another layer of computer between us. [Physician, internal medicine]
Seventy-five percent of my patients were Spanish speakers, and [the product didn’t support] Spanish speakers… [Physician, Internal Medicine]
I worry about my vulnerable patients. Like, will this help them? [Physician, internal medicine]

Abbreviations: AI, artificial intelligence; EHR, electronic health record.

Clinicians similarly valued the potential of ambient documentation, seeing ambient documentation as a tool to reduce burnout and allow more time for patient engagement, particularly improving nonverbal communication, such as eye contact (Box 2). Many were hopeful that the technology could help them synthesize medical data and improve their medical decision-making (Box 2). Some noted that verbalizing their thoughts during visits improved transparency, while others found value in sharing ambient documentation–generated notes with patients in real time, fostering a collaborative documentation process (Box 2).

Despite these benefits, both groups expressed concerns about data security and access. Common questions included: “Who has access to recordings?” “How long are they stored?” and “What guarantees ensure privacy?” (Box 2). Clinicians also were concerned that reliance on AI-generated documentation might weaken critical thinking skills. In addition, they highlighted potential equity risks, particularly for nonnative English speakers (Box 2).

### Perceptions of Responsibility for Ambient Documentation Tools

Patients and clinicians were asked to identify who they believed was responsible for key aspects of ambient documentation: clinical note accuracy, medical decisions based on AI-generated data, and security breaches. Both groups agreed that clinicians bear primary responsibility for ensuring clinical note accuracy (Box 3). Although many patients also saw vendors as responsible for AI inaccuracies, they overwhelmingly viewed physicians as the ultimate decision-makers when it came to any medical decisions made based on AI-generated notes; when asked about responsibility for medical errors linked to ambient documentation, 66 patients (64.1%) held physicians accountable compared with 25 patients (24.3%) who cited vendors and 11 patients (10.7%) who cited hospitals. For data security breaches, most patients (79 [76.7%]) believed vendors should be responsible, while fewer held hospitals (17 [16.5%]) or physicians (6 [5.8%]) accountable.

Box 3. **Representative Quotes for the Themes of Perceptions of Responsibility for Ambient Tools and Improving the Consent Process**

**Perceptions of responsibility for ambient tools**

It’s all on the doctor to make sure this is safe and accurate. [Patient]
I would hold my doctor accountable for any errors in my records or in my doctor’s diagnoses caused by ambient AI. [Patient]
If we are the ones who are using this, then the onus is on us as the doc to make sure things are accurate. [Physician, internal medicine]

**Improving the consent process—challenges**

Can I opt out or is this the way your office will be proceeding for now on? [Patient]
When you are counting every second of a visit, a consent plus an explanation is a waste of time. [Physician, internal medicine]
I don’t need to be involved with consent. You’re giving me more work to do. It doesn’t feel good. I don’t want to have to go through consent. When I take consent, it is different than when the clerk has consent. [Physician, medicine subspecialty]
In the moment, power dynamics are not appropriate—it’s hard to do great consent without an elaborate conversation, and time constraints interfere with such a conversation. [Physician, internal medicine]
Who wants to say no to their doctor to their face? The alternative is adding a consent form before the appointment but that has shortfalls too—people sometimes just agree without reading or miss it and then miss out on benefits. [Physician, internal medicine]
It should take place before the visit but that is also hard because if you ask patients at their check-in that is also leading some patients to answer yes or no based on their health literacy, demographics, etc. [Physician, internal medicine]
A common response is “Oh yeah, go ahead,” but another response is “Why do you have to ask me about this?” The asking in itself causes suspicion. [Physician, internal medicine, other]

**Improving the consent process—preferences and opportunities**

I would want the doctor to ask my consent before the exam begins. [Patient]
I would want to receive an email that my doctor/hospital/practice is using this the moment they begin using it. [Patient]
I would hope that there would be a website that I would have access to before my visit to provide me with more details and info about this tool. [Patient]
I think you should have the person at the front desk ask so the patient doesn’t feel pressured into saying yes. I would also state exactly where this recording is being sent to or who will have access to it. Then I would state the pros to using this AI. [Patient]
It’s probably better [to have a consent conversation] at the moment where you are pressing the [record] button, otherwise it feels sneaky somehow. [Physician, internal medicine]
The easiest thing would be to have everyone consent at registration. [Physician, internal medicine]
Not in the room when I’m there, something system-wide, maybe something at check-in. [Physician, internal medicine]
[Consent] should happen before the visit—it shouldn’t just be up to the clinician to get consent. It should be 2 processes, once when you confirm the appointment and then the doctor can address it again in the encounter. [Physician, internal medicine]
Should happen pre-visit, shouldn’t be just up to one person, the clinician, to get consent. Should be two processes, when you confirm the appointment, or yes, yes, I would like to learn more. And then doctor can address again in the encounter, just like should be the case with any consent process. [Physician, internal medicine]

Abbreviation: AI, artificial intelligence.

Although clinicians acknowledged their central role in overseeing the technology’s use, they were more likely than patients to view responsibility as being shared among several entities: individual clinicians, hospital systems, and the ambient documentation vendors. Similar to patients, clinicians agreed they had the final say on clinical note content, even when using AI tools (Box 3). However, they also emphasized the role of hospital legal and information technology teams in vetting these technologies. Clinicians and patients were aligned in viewing vendors as primarily responsible for data security, followed by the health system.

### Optimizing the Ambient Documentation Consent Process

Review of public records (eg, research documents, press releases, and white papers) of health care organizations’ approach to obtaining consent to use ambient documentation tools identified the most common method for obtaining consent as clinician-obtained verbal approval from patients at the time of the visit, followed by written consent forms. Review of ambient documentation vendor material largely deferred consent processes to the hospital system.

Both clinicians and patients saw room for improvement in the consent process. Clinicians cited time constraints, concerns about bias in presenting the technology, and uncertainty about what details to include (Box 3). Some questioned why consent was necessary because the tool was integrated into documentation practices (Box 3). Patients wanted more information, particularly regarding data use, storage, and access. Nearly all patients rated details around how audio was used (99 [96.1%]), where audio was sent (99 [96.1%]), and who had access to recordings (101 [98.1%]) as important or very important in their decision to consent. Many patients wanted a clear opt-out option and had questions regarding future withdrawal of consent.

Although both parties agreed the consent process could be improved, they were mixed on the best process. Most wanted a flexible approach that allowed for multiple touchpoints ahead of the visit to obtain information and ask questions prior to consenting (Box 3). Some clinicians preferred having the consent conversation immediately before activating the recording, believing it would feel more transparent. Other clinicians preferred that consent be obtained at check-in, to offload time burdens from clinicians and allow patients to opt out if uncomfortable. Many suggested a digitally enabled consent solution that leverages patient portal features to provide information and obtain consent prior to the visit. Patients also endorsed easy opt-out processes.

## Discussion

### Overview of Findings

This study explores clinician and patient experiences with informed consent for ambient documentation, examining current processes, limitations, and informational needs. Several factors were associated with patient consent: comfort with the technology, trust in the patient-clinician relationship, perceived risks and benefits, and responsibility for its use. Patients were generally more accepting of ambient documentation for routine visits than for sensitive discussions and preferred its use for documentation support over diagnostic reasoning. Data privacy and security concerns were the primary risks cited, with uncertainty about storage, access, and misuse often exacerbated by clinicians’ varied knowledge of data practices. Both parties agreed that physicians are responsible for note accuracy and decisions, while vendors are accountable for data breaches. Despite concerns, benefits included improved records, communication, and patient-clinician rapport. Both groups saw a need for consent process improvements—clinicians cited time constraints and power dynamics, while patients wanted more information and flexibility. A proposed 2-step consent process, involving digitally supported previsit notifications and in-visit confirmation, along with flexible withdrawal and opt-out options, was viewed as a potential solution.

### Findings in Context

To our knowledge, this is the first article to describe and evaluate the pragmatic experiences of patients and clinicians engaging in conversations around the use of AI-enabled ambient documentation technology. Consent emerged as a shared area of concern for both patients and clinicians, highlighting the process as a critical antecedent to the technology’s acceptance in health systems. Findings from this study reflect insights from other work in AI applications—some in ambient documentation but largely in other technology areas, such as computer vision and sensor data to health care. In a recent large-scale follow-up evaluation of ambient AI’s role in the Kaiser Permanent system in California, Tierney et al^13^ reported high satisfaction across more than 2.5 million users, with extremely low patient refusal rates (<0.5%) when standardized notification procedures—such as signage or verbal explanation—were in place. Duggan et al^14^ similarly found reductions in after-hours work but noted variability in how and when clinicians informed patients, with many unsure about consent requirements. In a response to the article by Duggan et al,^14^ Cohen et al^15^ outline key legal and ethical concerns surrounding the use of ambient listening AI, including patient consent; the authors argue that clinicians and health systems must obtain informed, meaningful consent from patients, going beyond generic disclosure to ensure patients understand what ambient recording entails, how their data will be used, and their right to opt out. They emphasize that some encounters (eg, discussion of intimate partner violence) may be inappropriate for the technology’s use and that data mismanagement could result in significant liability. Elsewhere, in a review of the ethical issues in “ambient intelligence” using sensors and wearables, Martinez-Martin et al highlight that the application of such devices coupled with machine learning algorithms to collect and interpret data raises issues of “privacy, data protection, informed consent, and fairness that might not be easily addressed through existing ethical and regulatory frameworks”^16^^(pe115)^; they note that use of ambient intelligence raises concerns about general surveillance technology in society and that even after informed consent is obtained patients may benefit from periodic visual or other reminders that recordings are taking place. Park^17^ identified high levels of support for consent among patients interacting with AI-supported diagnostic tools, despite the lack of ethical and legal consensus regarding whether disclosing use of medical AI is required for informed consent in many countries. These findings across studies mirror recommendations from medical ethics literature^18,19^ for health care systems to proactively engage patients in education and conversation about AI-enabled care delivery, even in scenarios where there is no legal requirement to disclose its use; this is seen as an opportunity for trust building and to promote technology acceptance among patients.^19^ This also aligns with broader efforts in the field of medical AI to promote explainability, transparency, and fairness and reduce unintended social harms around trust, surveillance, and discrimination.^20,21,22,23^

### Improving Consent

The integration of ambient AI into health care offers opportunities to reduce clinicians’ administrative burdens, but ethical challenges must be addressed to maintain patient trust and prevent disparities. Our research suggests that a comprehensive, multistep informed consent process is ideal to ensure the ethical use of a complex, patient-facing, AI-enabled technology, such as ambient documentation; we recommend that organizations use an opt-in approach, with clear communication about the technology’s capabilities and limitations, including detailed information about data privacy and security and its proposed uses in medical decision-making and care delivery, as well as clearly described constraints around withdrawal of consent. Consent should also include information on vendor partnerships and the specific responsibilities of the clinician and health systems vs those of the vendor. Patients should be informed about the use of ambient AI both before their visit and immediately prior to the clinical encounter, allowing them time to make a truly informed decision about whether to consent to its use (or to change their mind). In some instances, a partial opt in might also be considered, where patients can agree to ambient AI being used in certain contexts (eg, routine visits) but not others (eg, mental health assessments). Although inclusion of clinicians in the consent process was valued by both patients and clinicians, competing priorities during short visit times and pressure to outsource obtaining consent to nonclinical staff need to be considered; it may be appropriate to shift preliminary conversations to earlier touchpoints in the patient journey (eg, front desk or via the patient portal) while creating secondary moments with clinicians for additional conversation. Finally, given the unique and rapidly evolving nature of ambient AI technology, at this time, we suggest that consent to its use should remain separate from consent procedures for other types of technology use (eg, general patient consent to treatment forms).

### Strengths and Limitations

The study has several strengths, including its near-real-time data collection, as ambient AI technologies are deployed in clinical practices, and its inclusion of both diverse specialties (eg, primary care, medicine and surgical subspecialties) and patients across race and ethnicity and age groups. Despite this, there are also several limitations. The sample size was limited to a small number of clinicians and patients exposed to ambient documentation technology within our health system in the New York metropolitan area, which may not be representative of the broader population of either clinicians or patients and therefore limits generalizability. The sample was restricted to English-speaking participants and had a high proportion of individuals with a college or postgraduate education (81.6%), which may further limit representativeness. The reliance on self-reported information introduces the risk of recall bias from both patients and clinicians. Finally, the study does not directly analyze potential confounding variables, such as comorbidities, socioeconomic status, or social and structural determinants of health, which may influence perspectives and experiences with both the health care system and health-related technology; as a qualitative descriptive study, we are unable to make direct comparisons or identify causality in any of the findings.

## Conclusions

In this observational quality improvement study of patients and clinicians using AI-driven ambient documentation tools, informed consent emerged as a critical yet complex component of successful implementation. Participants highlighted the importance of trust, technical understanding, and communication in shaping comfort with these technologies. Although ambient documentation offers the potential to reduce clinician burden and enhance care delivery, its ethical integration depends on consent processes that are transparent, flexible, and inclusive. By adopting a comprehensive approach to informed consent and actively addressing the potential risks and benefits of these technologies, health care systems can ensure that ambient documentation enhances, rather than detracts from, the quality of patient care. Ongoing research and ethical analysis will be crucial in guiding the development of technologies that serve the interests of patients and clinicians.

## Notes

Supplement 1. **eAppendix 1.** Clinician Interview Questions **eAppendix 2.** Patient Survey Questions

Supplement 2.
Data Sharing Statement
