# Supplementary material for: Informed Consent for Ambient Documentation Using Generative AI in Ambulatory Care
Source: JAMA Netw Open. 2025 Jul 22;8(7):e2522400. doi: 10.1001/jamanetworkopen.2025.22400 (PMC12284739; doi:10.1001/jamanetworkopen.2025.22400)
Supplement: Supplement 1. — eAppendix 1. Clinician Interview Questions eAppendix 2. Patient Survey Questions [file jamanetwopen-e2522400-s001.pdf]

Supplementary Online Content

Lawrence K, Kuram VS, Levine DL, et al. Informed consent for ambient documentation using generative AI in ambulatory care. *JAMA Netw Open.* 2025;8(7):e2522400. doi:10.1001/jamanetworkopen.2025.22400

eAppendix 1. Clinician Interview Questions

eAppendix 2. Patient Survey Questions

This supplementary material has been provided by the authors to give readers additional information about their work.

© 2025 Lawrence K et al. *JAMA Network Open.*

eAppendix 1. Clinician Interview Questions

1. How do you talk with patients about the use of AMBIENT?
2. How have your patients responded to your use of AMBIENT?
    a. Are there any anecdotes or questions that patients shared that stood out to you? Why?
3. What is your brief overall perception of AI technology?
4. What is top of mind for you thinking about the use of AI in healthcare?
    a. [Prompt] What comes to mind when thinking about ethical considerations for AI in healthcare?
5. Now that you've used AMBIENT for a bit, tell me what impact it has on the clinical encounter.
    a. [Prompt] What feels different? Better? Worse?
6. What impact do you think AMBIENT has had on the relationship with your patients?
    a. [Prompt Is AMBIENT a net positive introduction? Net negative? Why?
    b. [Prompt] What impact, if any, does AMBIENT have on your ability to establish trust with your patients?
7. What do you think of the current process of asking patients to consent to using AMBIENT? Why?
    a. At what point in the patient's journey should we obtain consent for products like AMBIENT? Options...
    b. Based on where you just said consent should happen, what would that ideal process look like?
8. What information do you wish you had about the AMBIENT product?
    a. [Prompt] Do you wish you had more information about any of the following: data storage and usage, model training, legal responsibility for issues, something else?
9. Is there anything else I should have asked you about AI health tech and ethics?

© 2025 Lawrence K et al. *JAMA Network Open.*

eAppendix 2. Patient Survey Questions

1. Tell us about your relationship with your doctor.

2. How much do you agree with the following statement? “*I trust my doctor.”*
    - Strongly Disagree
    - Disagree
    - Neutral
    - Agree
    - Strongly Agree
3. How much do you agree with the following statement? “*I feel respected by my doctor.”*
4. How much do you agree with the following statement? “*My doctor pays attention to me during our visit.”*
5. How much do you agree with the following statement? “*I feel heard by my doctor."*
6. How much do you agree with the following statement? “*My doctor explains medical decisions well*.”
7. How much do you agree with the following statement? “*My secrets are safe with my doctor.”*

8. Tell us about your recent experiences using AMBIENT technology with your doctor. As a reminder, this is the technology that records your conversation with your doctor and helps them write a note in the medical record. What was it like?

9. How comfortable do you feel agreeing to let your doctor use this technology?
    - very uncomfortable
    - somewhat uncomfortable
    - Neutral
    - somewhat comfortable
    - very comfortable

10. What made you feel that way about letting your doctor use this technology?
11. What other questions do you have for your doctor at this time?

12. Imagine that the next time you visit your doctor, they greet you with this question: “*Hello. Before we start, I’m using a new technology that helps me take notes by recording our visit. It allows me to be more efficient and focus better on you. Would that be ok?”* What would you do in this situation?
    - Answer “Yes”

© 2025 Lawrence K et al. *JAMA Network Open.*

- Answer “No”
- Recommend my doctor to others for embracing this tech
- Censor or edit what I say to my doctor
- Leave my doctor's office
- Ask a follow-up question (tap to type)

13. We would now like to give you more information about this technology: “Your doctor is using a technology called Ambient AI, which audio records your visit and uses artificial intelligence (AI) technology to automatically summarize your visit into a format that is easy for your doctor to review. Your doctor can use this summary to remember your visit and make decisions about your care. This technology often works very well but can also make errors. Your doctor uses this technology in partnership with a for-profit technology company.” What would you do in this situation?
    - Answer “Yes”
    - Answer “No”
    - Recommend my doctor to others for embracing this tech
    - Censor or edit what I say to my doctor
    - Leave my doctor's office
    - Ask a follow-up question (tap to type)

14. With this additional information, how comfortable would you feel agreeing to let your doctor use this technology?
    - very uncomfortable
    - somewhat uncomfortable
    - Neutral
    - somewhat comfortable
    - very comfortable

15. What would you do if your doctor used Ambient AI and wanted to discuss your mental health during the visit?
    - I would leave the practice or not see my doctor again
    - I would censor what I say to my doctor
    - No change in my behavior
    - I would have a better or more open conversation with my doctor
    - I would recommend my doctor for embracing this technology
16. What would you do if your doctor used Ambient AI when doing a routine physical?

© 2025 Lawrence K et al. *JAMA Network Open.*

17. What would you do if your doctor used Ambient AI when talking through a death in the family?
18. What would you do if your doctor used Ambient AI when receiving a difficult diagnosis?
19. What would you do if your doctor used Ambient AI when discussing abuse?
20. What would you do if your doctor used Ambient AI when talking about illicit, illegal, or compromising activities?
21. What would you do if your doctor used Ambient AI when discussing your sexual health during the visit?

22. How comfortable do you feel with your doctor using Ambient AI to summarize the content of your conversation?
    - very uncomfortable
    - somewhat uncomfortable
    - Neutral
    - somewhat comfortable
    - very comfortable
23. How comfortable do you feel with your doctor using that AI-generated summary to come up with a treatment plan?
24. How comfortable do you feel with your doctor using that AI-generated summary to diagnose you with a disease?
25. How comfortable do you feel with your doctor sharing your recording with the technology company to improve their product?

26. How important is it that you know the answer to the following question before allowing your doctor to use Ambient AI during your visit: “Where does the audio of my visit go?"
    - Not at all important
    - Unimportant
    - Neutral
    - Important
    - Very important
27. How important is it that you know the answer to the following question before allowing your doctor to use Ambient AI during your visit: “How is the audio from of my visit used?”
28. How important is it that you know the answer to the following question before allowing your doctor to use Ambient AI during your visit: “Who has access to the audio of my visit?”

© 2025 Lawrence K et al. *JAMA Network Open.*

29. How important is it that you know the answer to the following question before allowing your doctor to use Ambient AI during your visit: “*Will I get in trouble for any sensitive, secret, or illegal activities discussed in the audio?”*

30. Who do you think is the most responsible for fixing the following problem? *The AI-generated visit summary is inaccurate.*
    - Doctor
    - Hospital System
    - Ambient AI Company
    - Yourself
    - Someone else
31. Who do you think is the most responsible for fixing the following problem? *You receive an incorrect diagnosis based on inaccurate AI-generated content.*
32. Who do you think is the most responsible for fixing the following problem? *Your health information is leaked by a hacker of the Ambient AI company.*
33. Who do you think is the most responsible for fixing the following problem? *Ambient AI cannot understand your accent or language.*

34. When would you want your doctor to tell you about Ambient AI and ask for permission to use the technology during your visit?
    - Informed and asked in a text message or call a few days before your appointment
    - Informed and asked by staff at the check-in desk the day of your appointment
    - Informed and asked by your doctor at the start of your appointment
    - Informed after your visit that Ambient AI was used; asked if you’d like to continue use for future appointments
    - Other (tap to type)

35. Is there anything else you would like to add about your perceptions of Ambient AI?
36. Anything we should have asked you that we didn't

© 2025 Lawrence K et al. *JAMA Network Open.*
