# Supplementary material for: Informed Consent for Ambient Documentation Using Generative AI in Ambulatory Care
Source: JAMA Netw Open. 2025 Jul 22;8(7):e2522400. doi: 10.1001/jamanetworkopen.2025.22400 (PMC12284739; doi:10.1001/jamanetworkopen.2025.22400)
Supplement: Supplement 2. — Data Sharing Statement [file jamanetwopen-e2522400-s002.pdf]

Data Sharing Statement

Lawrence. Informed Consent for Ambient Documentation Using Generative AI in Ambulatory Care. *JAMA Netw Open*. Published July 22, 2025. doi:10.1001/jamanetworkopen.2025.22400

Data
Data available: No

Additional Information
Explanation for why data not available: Data on individual clinicians and patients are not publically available as they may contain sensitive identifiable information. De-identified study data may be made available by request.
